# An advanced molecular medicine case report of a rare human tumor using genomics, pathomics, and radiomics

**DOI:** 10.3389/fgene.2022.987175

**Published:** 2023-02-10

**Authors:** Li Ma, Erich A. Peterson, Ik Jae Shin, Jason Muesse, Katy Marino, Mathew A. Steliga, Omar Atiq, Konstantinos Arnaoutakis, Christopher Wardell, Jacob Wooldridge, Fred Prior, Donald J. Johann

**Affiliations:** ^1^ Winthrop P. Rockefeller Cancer Institute, University of Arkansas for Medical Sciences, Little Rock, AR, United States; ^2^ Department of Information Science, University of Arkansas at Little Rock, Little Rock, AR, United States; ^3^ Department of Biomedical Informatics, University of Arkansas for Medical Sciences, Little Rock, AR, United States; ^4^ Department of Radiology, University of Arkansas for Medical Sciences, Little Rock, AR, United States

**Keywords:** pulmonary sclerosing pneumocytoma, molecular profiling, TP53 signaling pathway, genomics, radiomics, pathomics, case report

## Abstract

**Background:** Pulmonary Sclerosing Pneumocytoma (PSP) is a rare tumor of the lung with a low malignant potential that primarily affects females. Initial studies of PSP focused primarily on analyzing features uncovered using conventional X-ray or CT imaging. In recent years, because of the widespread use of next-generation sequencing (NGS), the study of PSP at the molecular-level has emerged.

**Methods:** Analytical approaches involving genomics, radiomics, and pathomics were performed. Genomics studies involved both DNA and RNA analyses. DNA analyses included the patient’s tumor and germline tissues and involved targeted panel sequencing and copy number analyses. RNA analyses included tumor and adjacent normal tissues and involved studies covering expressed mutations, differential gene expression, gene fusions and molecular pathways. Radiomics approaches were utilized on clinical imaging studies and pathomics techniques were applied to tumor whole slide images.

**Results:** A comprehensive molecular profiling endeavor involving over 50 genomic analyses corresponding to 16 sequencing datasets of this rare neoplasm of the lung were generated along with detailed radiomic and pathomic analyses to reveal insights into the etiology and molecular behavior of the patient’s tumor. Driving mutations (AKT1) and compromised tumor suppression pathways (TP53) were revealed. To ensure the accuracy and reproducibility of this study, a software infrastructure and methodology known as NPARS, which encapsulates NGS and associated data, open-source software libraries and tools including versions, and reporting features for large and complex genomic studies was used.

**Conclusion:** Moving beyond descriptive analyses towards more functional understandings of tumor etiology, behavior, and improved therapeutic predictability requires a spectrum of quantitative molecular medicine approaches and integrations. To-date this is the most comprehensive study of a patient with PSP, which is a rare tumor of the lung. Detailed radiomic, pathomic and genomic molecular profiling approaches were performed to reveal insights regarding the etiology and molecular behavior. In the event of recurrence, a rational therapy plan is proposed based on the uncovered molecular findings.

## 1 Introduction

Pulmonary Sclerosing Pneumocytoma (PSP) is a relatively uncommon benign tumor of the lung with potential for malignant transformation that is manifested most commonly by metastasis to regional lymph nodes (Zheng et al., 2022). PSP was first reported by Liebow in 1956 (Liebow and Hubbell, 1956), and shows a striking female predominance (female to male ratio 5:1) (Kalhor et al., 2010). Histologically, PSP is primarily composed of 2 cell types (cuboidal epithelial and polygonal stromal cells) and four histological types (hemorrhagic, sclerotic, solid and papillary) (Gao et al., 2020).

Due to the lack of noteworthy clinical or imaging findings, PSP is hard to recognize, and most cases are diagnosed by histopathological analysis (Song et al., 2021). The neoplasm may be confused with other benign nodules like hamartoma, tuberculoma, bronchial cysts, or certain lung cancers (Cheung et al., 2003). Often, patients are asymptomatic and PSP is detected incidentally. Non-specific associated symptoms may include: cough, chest pain, chest tightness and hemoptysis (Cardemil et al., 2004).

Initial studies of PSP focused primarily on analyzing features discovered using conventional X-ray or CT imaging. PSP has been described as a distinct, juxta-pleural nodule with strong and homogeneous enhancement on CT (Im et al., 1994; Xie et al., 2003). Nevertheless, using the above-mentioned techniques, there are no specific or classic imaging findings associated with PSP (Wang et al., 2011).

In recent years, because of the widespread use of next-generation sequencing (NGS), the study of PSP at the molecular-level has emerged. PSP lacks the classic driver gene mutational signatures of lung adenocarcinoma, e.g., EGFR, KRAS; ALK, or ROS1 fusions (Sartori et al., 2007; Pal and Chetty, 2020). A study utilizing whole-exome sequencing to explore genomic modifications in PSP has been performed (Jung et al., 2016). That study confirmed a high frequency of AKT1 point mutations (overall 31 of 68 patients, 46%) including p.E17K. It has been postulated that AKT1 mutations are the genetic hallmark of PSP (Yeh et al., 2020). Another study revealed that irregular activation of the mTOR pathway is a consistent genetic event in PSP (Boland et al., 2021). The PI3K/AKT/mTOR pathway is one of the most frequently activated oncogenic pathways (Porta et al., 2014), and activated AKT phosphorylates mTOR, which activates mTORC1.

This is the first study to use an advanced quantitative molecular medicine approach to provide a more thorough description of PSP. Using a combination of genomics, radiomics (Lambin et al., 2017) and pathomics (Gupta et al., 2019) a comprehensive description of the patient’s presentation as well as the molecular determinants of this rare tumor are provided along with a precision medicine therapy plan in case of recurrence.

## 2 Case presentation

The patient is a pre-menopausal female who was admitted to the hospital because of progressive and severe left sided flank pain over a 1-week duration. The patient was a former smoker (cigarettes, one pack/day) for 7 years, who quit 2 years ago. She currently uses vaping products on a regular basis. The initial clinical suspicion included a possible kidney stone; however, imaging studies were negative for stones, but did reveal a 3 cm mass in the left lower lung. Following a referral to medical oncology a lobectomy of the left lower lung for curative intent was performed by thoracic surgery. Histopathologic features were consistent with pulmonary pneumocytoma cell types, the tumor measured 3.2 cm in greatest dimension, surgical margins were clean, and two hilar/peribronchial lymphnodes were negative for malignancy (stage Ib, p.T2a.N0.M0, NCCN v.3.2022). Also identified were abundant hemosiderin-laden macrophages, compatible with vaping related lung injuries.

## 3 Methods

### 3.1 Ethical compliance

This study is part of a clinical trial (NCT02597738) approved by the Institutional Review Board of the University of Arkansas for Medical Sciences (UAMS). As part of this trial, written informed consent was obtained from the patient for research use of clinical specimens and associated data.

### 3.2 Genomics sample preparation

The QIAGEN QIAseq Human Lung Cancer Panel (DHS-005Z) library prep kit (QIAGEN, 2022) was used for targeted DNA-based assays involving tumor and normal (T/N). Supplementary File 1 in BED format contains the exact regions of interest for the amplicon-based assay. An Illumina HiSeq 3000 was utilized for all NGS studies. The lung cancer panel, which utilizes uniform molecular identifiers (UMIs) was run with a coverage of 3,000x for the tumor and 600x for the germline. Whole genome sequencing (WGS) libraries were constructed using the New England BioLabs (NEB) NEBNext Ultra II DNA library prep kit (NEB, 2022), and sequenced in an ultra-low-pass fashion for copy number analysis (CNA) at ∼0.3x coverage for T/N. For RNA-based experiments, the Illumina TruSeq Stranded Total RNA library prep kit (Illumina, 2022) was used. Six biological replicates were utilized for the tumor and six for the normal adjacent lung tissue. Sequencing was targeted at 200M reads for these 12 samples. In summary, six biological replicates of the tumor and adjacent normal lung (12 RNA NGS libraries) were built and sequenced, and four DNA libraries were built and sequenced.

### 3.3 Genomics molecular profiling

Genomics datasets were processed as previously reported by the NGS Post-pipeline Accuracy and Reproducibility System (NPARS), a reproducible software infrastructure developed by our group (Ma et al., 2021). Three separate pathway analysis tools were utilized and all run using default parameters. For canonical signaling pathway analysis, two traditional pathway analysis tools were used, pathfindR v1.6.3 (Ulgen et al., 2019) and Gene Set Enrichment Analysis (GSEA) v4.2.3 (Aravind et al., 2005). Additionally, an unsupervised pathway analysis tool named Weighted Correlation Network Analysis (WGCNA) v1.71 (Langfelder and Horvath, 2008) was used and then limma (v3.52.1) based methods were employed to further elucidate outputs generated by WGCNA. A normalized RNA-seq gene counts matrix, which was generated by NPARS via DESeq2 v1.36.0 (Love et al., 2014), was used as input for signaling pathway analyses.

### 3.4 Radiomics

DICOM imaging studies from the initial medical workup were obtained from the UAMS PACS and converted to NIfTI format. Segmentations and visualizations were produced using 3D Slicer v4.13 (Fedorov et al., 2012). Tumor segmentations (performed via thresholding techniques) were produced from CT studies. The border region was segmented by adding a margin of 10 mm to the tumor. Radiomic features were extracted from original images using Pyradiomics (van Griethuysen et al., 2017), both in aggregate for segmentations and as feature maps. A bin width of 25 voxels was used, and feature maps used a kernel radius of 1 voxel and calculated in 2D space. The entropy radiomics feature used is defined by the Image Biomarker Standardization Initiative as intensity histogram entropy (Zwanenburg et al., 2020).

### 3.5 Pathomics

Whole slide images were acquired using an Aperio CS2 whole slide imaging scanner (Leica Biosystems) at ×40 magnification. Image analysis was performed using the open-source program QuPath (v0.3.2) that included a suite of tools (Bankhead et al., 2017). Representative areas of the slide were annotated by a pathologist, indicating areas of tumor, hemosiderin-laden macrophages, and background lung parenchyma. From these areas, cell nuclei were segmented using StarDist with the *he_heavy_augment* model as described in the QuPath documentation (Schmidt et al., 2018). Cell expansion was enabled to approximate overall cell size. Cell classification was accomplished using the built-in object classifier to train a random trees classifier using the default feature extractor. Features included measurements of area, shape and, color of nuclei, cytoplasm, and overall cell.

## 4 Results

**FIGURE 1:**
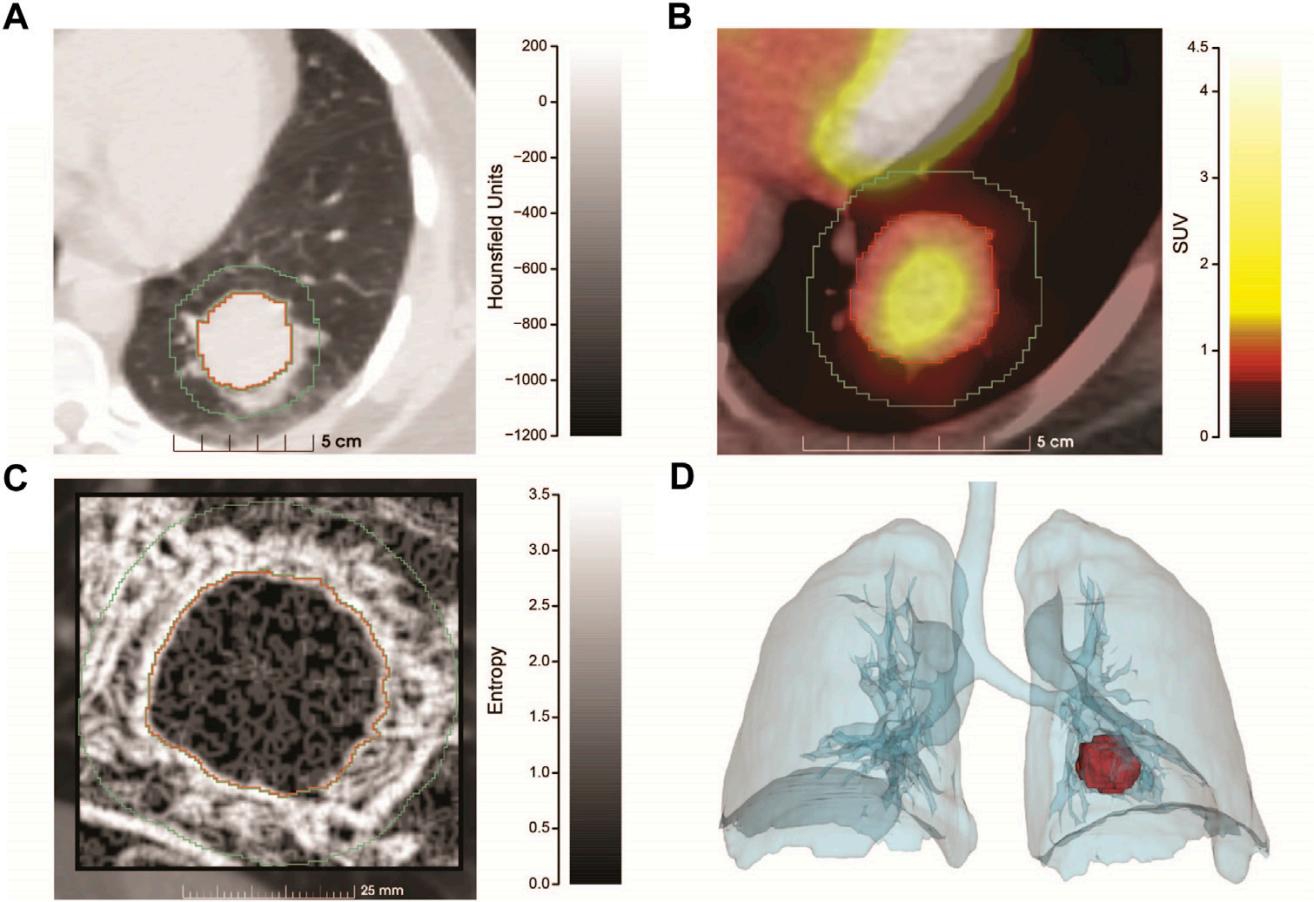
Radiomics analysis of the PSP tumor. **(A)** Pre-operative chest CT scan with contrast utilizing lung window settings. The image is an axial projection that has been zoomed to show an optimal view of the tumor that resides in the left lower lung along with a small region of the mediastinum. Tumor segmentation is outlined in red, with the 1 cm border surrounding the tumor proper, outlined in green. The x-axis contains a size scale (cm) and y-axis Hounsfield Units (HU) scale (−1200–200) with shading. **(B)** Combined PET/CT of the tumor (zoomed) at diagnosis. The tumor had a SUV max of 2.2 and SUV mean of 1.3, the x-axis contains a size scale (cm) and y-axis contains the SUV scale (0–4.5) with color coding. **(C)** Feature map showing the entropy of the tumor and 1 cm surrounding region, generated from a sagittal slice of the CT at presentation. The tumor is significantly more homogenous than the surrounding region. The x-axis contains a size scale (mm) and y-axis contains an entropy scale (0–3.5) with shading. **(D)** Volume rendering showing the size and position of the tumor at diagnosis. Produced using segmentations of the lungs and tumor from the PET/CT series.

Figure 1 shows the salient medical imaging for the patient and results from radiomics analyses. Sub-image **(A)**, shows a pre-operative chest CT image with contrast, zoomed to show a more optimal view of the tumor in the left lower lung. Segmentations of the tumor and a 1.0 cm circumferential border were performed. At presentation, the tumor had a maximum diameter of 3.2 cm, minimum diameter of 2.8 cm and a volume of 19.6 cm^3^. The median radiodensity of the tumor was 41 HU, approximately midway between the median densities of the kidneys (24 HU) and the liver (58 HU). As reference, the median density of normal lung (alveolar space) is ∼ -650.

As part of cancer staging a PET/CT study (**B**) was performed. Raw PET values were converted to standardized uptake values (SUV). The mean SUV in the tumor was 1.3 with a maximum SUV of 2.2. A reference volume of approximately 3 cm was measured in the liver (standard comparison), which had a mean SUV of 1.2 and maximum SUV of 1.5, implying that the tumor had relatively low metabolic activity.

From the CT study, radiomic features were extracted (**C**) and compared between the tumor and surrounding border region representing the tumor microenvironment. Radiomic features are most informative when comparing many similar tumors, but salient information can be inferred from a single case. We extracted the entropy of the segmentations (**C**), which is a measure of the amount of information required to encode the voxels of the image. Entropy measures the randomness of the voxel values, where low values represent more homogenous regions and higher values represent more heterogenous regions. The median entropy of the tumor and border regions were 0.92 and 1.89 respectively, illustrating that the microenvironment (border region) was more complex (heterogeneous). This result was highly statistically significant using a two-sided Wilcoxon test (*p* < 2.2 x 10^−16^). Finally, volume rendering showing the size and position of the tumor (**D**) was produced using segmentations of the lungs and tumor from the PET/CT study.

**FIGURE 2:**
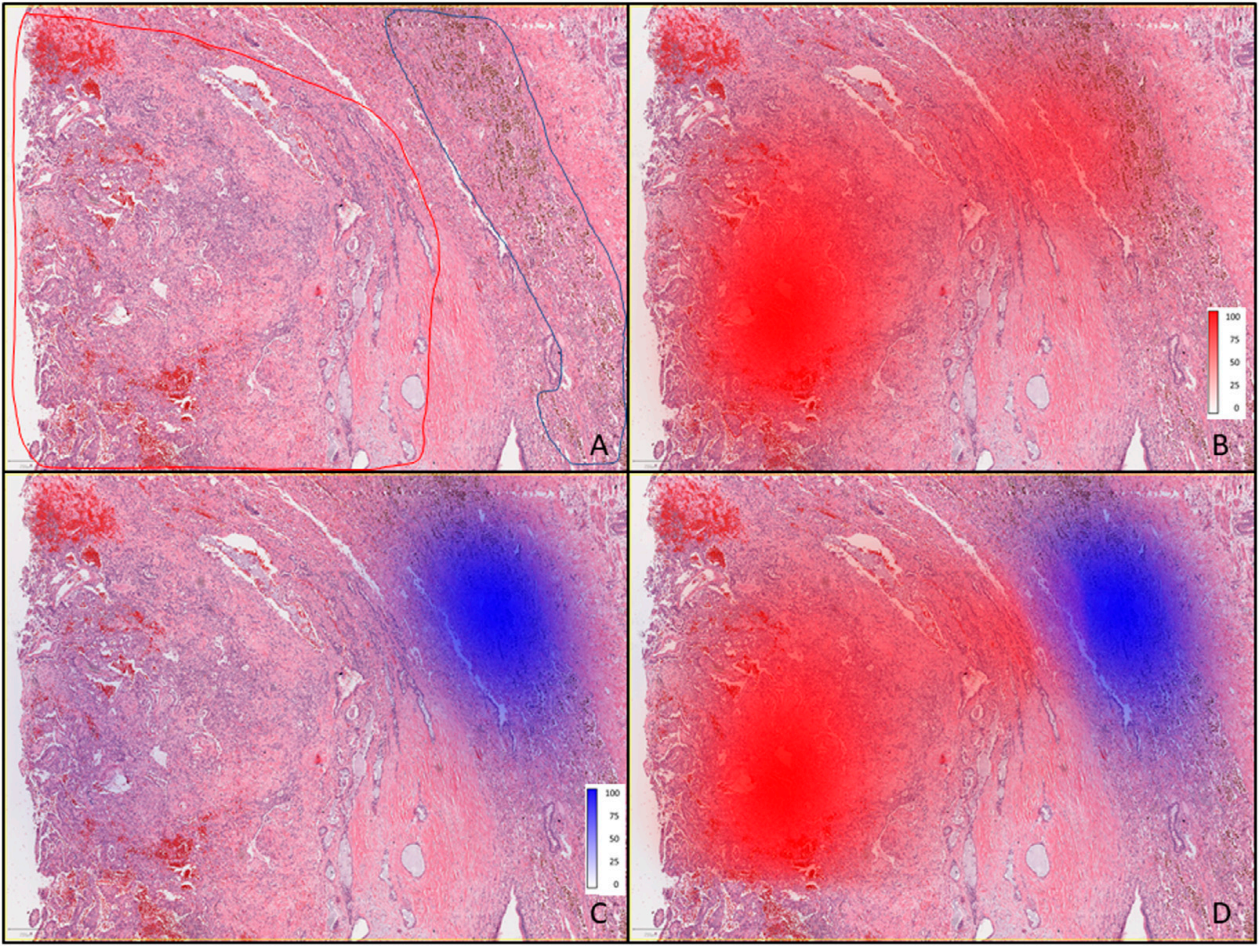
Pathomics analysis of the PSP tumor. **(A)** Low-power (4×) photomicrograph showing areas containing tumor (outlined red) and hemosiderin-laden macrophages (outlined blue) as annotated by the pathologist. **(B)** Tumor with red color density maps showing the number of cells per mm^2^ as identified by the classifier, and shown as percentages (0–100), where the 100% scale value corresponds to 1660 cells per mm^2^, along with intense red coloring. **(C)** Tumor tissue with blue color density maps showing hemosiderin-laden macrophages where the most intense blue color and scale value of 100% corresponds to 349 cells per mm^2^ (as identified by the classifier). **(D)** Overlaid density maps for both cell types (same classifier results and color intensity scales as in **(B,C)**.

Figure 2 displays the results of pathomics analyses. As background, nuclear segmentation using StarDist performed well overall, with the primary deficiencies being occasional segmentation of large cytoplasmic blebs without a visible nucleus, over-estimation of nuclear size in foamy macrophages, and difficulty distinguishing nuclei from hemosiderin in some hemosiderin-laden macrophages. In Supplementary Figure 1, examples of measurement maps corresponding to cell circularity are shown overlaid onto intermediate magnification photomicrographs of background lung and the tumor. In Figure 2, the pathologist’s annotations (**A**) are shown in a low-power (4x) photomicrograph for areas containing tumor (red) and hemosiderin laden macrophages (blue). Density maps for cells classified as tumor (**B**), and as hemosiderin-laden macrophages (**C**) for a region of tissue which was not used for classifier training are displayed separately and then jointly (**D**).

**FIGURE 3:**
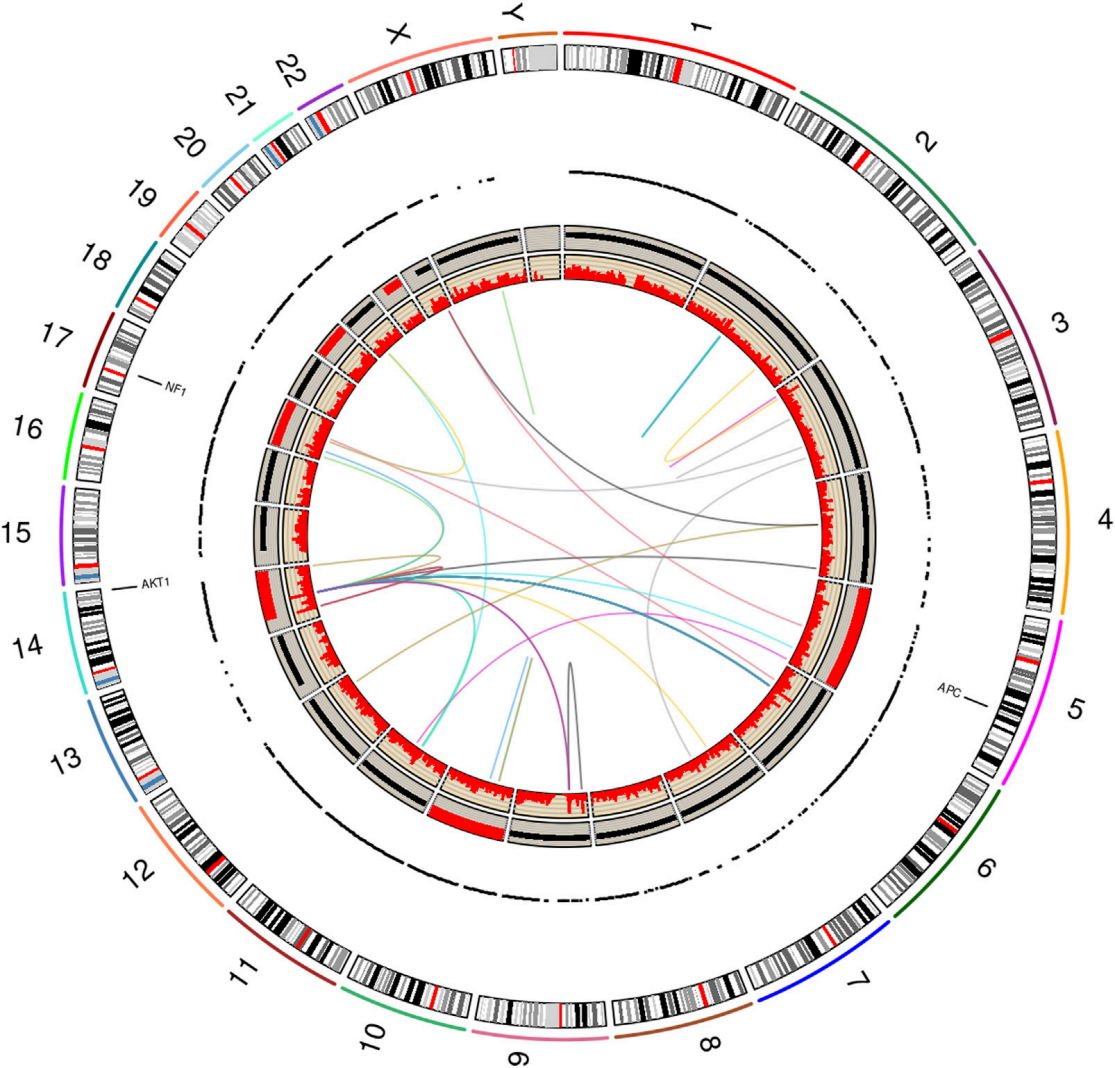
RCircos plot produced by the NGS Post-pipeline Accuracy and Reproducibility System (NPARS). This figure summarizes and integrates seven genomics methods into one graphical plot. From the outermost ring inward: (i) human chromosomal ideogram, (ii) DNA panel mutations (tumor vs. germline), (iii) RNA expressed mutations from the full transcriptome, each dot represents a RNA expressed mutation (depth greater than or equal 10), (iv) whole genome DNA copy number variations (tumor vs. germline) with red representing a copy number greater than 2, copy number equal to 2 by black coloring, and a copy number smaller than 2 by blue, (v) RNA gene expression (TPM) and, (vi) RNA gene fusions.

Figure 3 displays a graphic produced by RCircos v1.2.2 (Zhang et al., 2013), which summarizes and integrates the findings of seven genomics methods into a single graphical image. The layout of the RCircos diagram is as follows, from the outmost circle inward this plot contains: i. human chromosomal ideogram, ii. lung cancer targeted 72 gene panel for T/N, iii. RNA expressed mutations from the full transcriptome (represented as a “dot” due to spacing), iv. WGS DNA T/N CNA with the red color representing amplification, black for normal, and deletion as blue, v. Tumor RNA gene expression and, vi. Tumor RNA gene fusions. In our study, 52 total genomic analyses were generated and analyzed, specifically: DNA targeted panel T/N, DNA ultra-low-pass WGS T/N for CNA, RNA studies involving six biological replicates from the tumor and the normal adjacent lung (12 samples) subjected to: 1) RNA expressed mutation analysis, ii) statistical inferencing with DESEq2 (Love et al., 2014), and iii) Fusion analysis via STAR-Fusion (Haas et al., 2019). Supplementary Figure 2 illustrates the tissue specimens and genomic analyses (total of 52) generated.

Examining Figure 3, three somatic non-synonymous DNA mutations were found by the targeted DNA panel: AKT1 p.E17K, NF1 p.H1826Y, APC p.V1822D, with sequencing depths of 6,243 (allelic frequency: 36.75%), 5,809 (6.72%), 9,735 (61.6%) respectively (see Supplementary Table 1 for targeted DNA panel details). The AKT1 mutation is a driver for PSP tumors (Yeh et al., 2020), the findings for NF1 and APC are not drivers. The germline TP53 mutation p.P72R was detected with a depth of 1573 and an allelic frequency of 50%, but this is not indicated to be of significance per ClinVar (TP53). Finally, a TP53 p.K382fs frameshift mutation was found at the low allelic frequency of 0.6% and a depth of 5158; however, the mutation did not pass filter by smCounter2 (Xu et al., 2019) (homopolymer).

Due to RNA-seq experiments covering the entire transcriptome and the use of six biological replicates, a total of 1,119,654 RNA expressed mutations were found to pass filter by HaplotypeCaller (DePristo et al., 2011; Van der Auwera et al., 2013). Using the recommended depth filter of 10 from Guo et al. and limiting mutations to those having a predicted impact of moderate or high, the RNA expressed mutation analysis was further filtered (Guo et al., 2017). After filtering, 8,139 mutations remained for further analysis. Among these mutations, 2,938 of them are found in all six tumor samples (see Supplementary Table 2), and 1,854 mutations are private to specific samples (see Supplementary Table 3). Based on the RNA-seq VCF files of the six tumor samples and the six normal samples, a phylogentic analysis was performed using PHYLIP v3.697 (PHYLIP) (see Supplementary Figure 3). The PHYLIP dendrogram shows a clear separation of tumor vs. normal and with the tumor arising from the normal. The driving mutation found in the DNA study, AKT1 p.E17K was expressed in five of six RNA biological replicates with a depth range of 101–471, and VAF range of 28%–49% (see Supplementary Table 4).

Ultra-low pass WGS experiments revealed copy number variations concentrated in chromosomes 5, 10, 14, 17, 19 and 21 (all amplifications). All the three DNA mutated genes, AKT1, NF1, and APC, were amplified (see Supplementary Table 5; Supplementary Figure 4). A differential gene expression (DGE) analysis was performed by DESeq2 (Love et al., 2014) on the RNA-seq data via NPARS. DGE analysis revealed 11,646 genes to be significantly differentially expressed (adjusted *p*-value < 0.1) between the tumor and matched normal adjacent lung replicates (see Supplementary Table 6). A significant finding was the overexpression of MDM2 in the tumor (log2 fold change: 1.33; q-value: 2.93E-11), a key regulator in the TP53 pathway.

RNA-seq gene fusion analysis showed a number of fusion events across the genome (see Supplementary Table 7), with TIMM23-PARGP1 found in all six tumor replicates. However, the TIMM23-to-PARGP1 fusion does not drive PSP, in the literature to-date. The total distinct fusions found across all six tumor replicates and passed by STAR-Fusion was 36.

**FIGURE 4:**
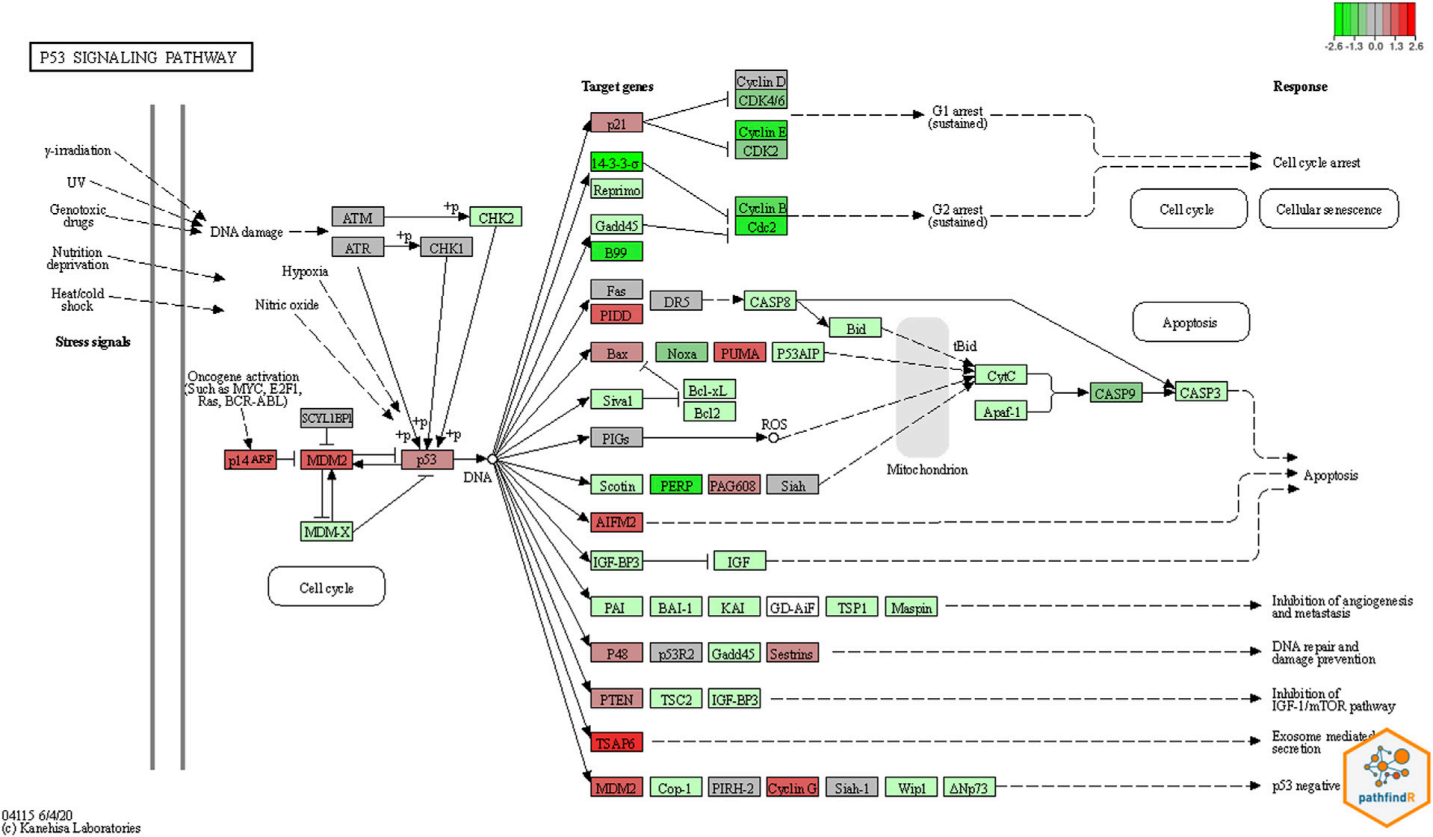
Pathway Result by *pathfindR*. The colored plot was generated based on KEGG pathway diagrams through *pathfindR*. Red colors represent upregulated genes, and green colors the down regulated genes. In this plot for the TP53 pathway, MDM2 which is the principal negative regulator of the pathway, was significantly upregulated.

Using RNA-seq data (tumor and normal adjacent lung biological replicates), both conventional signaling pathway analysis tools, pathfindR and GSEA, found a large number of abnormal candidate pathways. The pathways found to be statistically significant by pathfindR are listed in Supplementary Table 8. The GSEA’s most significant pathways are listed in Supplementary Table 9. WGCNA initially clusters genes into significant modules (in this study, there are total of 100 modules). Then using the R package limma v3.52.1, the most significantly differentiated modules were extracted (Ritchie et al., 2015). Next, the most differentiated module (module number 1, containing 5,108 genes), was sent to pathfindR for further analysis. The most significant pathways for genes within module number 1 were identified (see Supplementary Table 10). Comparing the output from these pathway analysis tools, we found that the TP53 signaling pathway to be statistically significant by all three pathway analysis tools, and MDM2 overexpressed. Using pathfindR’s KEGG (Kanehisa and Goto, 2000) integration, the TP53 pathway shown in Figure 4.

## 5 Discussion and conclusion

Why does a relatively young woman develop an unusual tumor in her lung? How is her presentation involving left flank pain related to her pathologic processes? Using genomics, radiomics and pathomics we sought to bring additional clarity to these questions.

The patient presented with severe left flank pain. It is established that disease processes or injuries involving the lower lung may present as flank pain (LeBlond, 2015). The 3D position of the tumor and the proximity to the left lung base is nicely displayed by the radiomics study in Figure 1D. Utilizing segmentation and entropy calculations (Figure 1C) radiomics showed the tumor region to be much more homogeneous vs. a surrounding 1 cm rim representing an inflamed microenvironment, which is now known to be filled with abundant hemosiderin-laden macrophages. Hemosiderin-laden macrophages are an important finding regarding an acute lung injury and indicates alveolar hemorrhage (Beasley, 2010). This finding was also observed and quantified by the pathomics study (Figures 2C,D). The patient’s lung injury is related to her vaping practices and may be manifested in left lower lung due to tumor growth and corresponding increased metabolism (Figure 1B).

The first principal genomic finding of this study, was the detection of the AKT1 p.E17K mutation within both the DNA and RNA of the patient’s tumor with convincing VAF and depth of coverage. This finding is consistent with previous studies that have shown many PSP cases to harbor AKT1 mutations (Jung et al., 2016; Yeh et al., 2020). There is a growing body of evidence that AKT1 mutations are a hallmark of PSP (Yeh et al., 2020), and this oncogene can be assumed to be the driving mutation for this patient’s tumor.

AKT1 is a member of the AKT kinase family. As meaningful down-stream regulators of the PI3K signaling pathway, members of the AKT kinase family play an import role. In all cancers, the PI3K/AKT pathway is considered one of the most frequently deranged (Mundi et al., 2016). Although our signaling study did not find the pathway to be statistically significant, the pathway contains a mutated AKT1, driving tumor proliferation (Yeh et al., 2020), and is a viable drug target.

The second principal genomic finding, was that the TP53 signaling pathway was found to be statistically significant in all three pathway analysis methods. Chief among the alteration of genes in the TP53 pathway is that the p53 inhibitor MDM2 is significantly over-expressed in the patient’s tumor. The overexpression of MDM2 in tumors inhibits p53 and favors an uncontrolled environment for cell proliferation (Chène, 2003; Hou et al., 2019). This helps to explain an additional reason for tumor development. Namely, a dampened response regarding tumor suppressor function by an essential pathway focused on tumor surveillance and eradication.

In the TP53 signaling pathway, p53 and MDM2 proteins form a central hub which is one of the key molecular complexes most frequently connected to other signaling pathways in the cell. The MDM2-p53 hub receives stress inputs, and by forming and changing a large number of other pathways and functions in the cell, p53 responds to the inputs (Levine, 2020). The MDM2-p53 hub is also a negative feedback loop. In this loop, MDM2 is transcriptionally induced by p53, but reciprocally blocks p53 activity (Zhou et al., 2017). According to the colored KEGG pathway plot generated by pathfindR (Figure 4), it is evident that the MDM2 gene is significantly upregulated.

Per standard-of-care guidelines, the patient had a lung surgery for curative intent, but a precision oncology therapy plan was formulated as a precaution in case of tumor recurrence. Active clinical trials enrolling patients that target MDM2 abnormalities and AKT1 p.E17K mutations exist. Regarding MDM2 inhibitors: (i) RO5045337 (Roche), prevents the MDM2 protein from binding to the transcriptional activation domain of p53 (NCI, 2022; Roche, 2022); (ii) siremadlin (HDM201, Novartis), increases the activity of the tumor suppressor p53 by selectively inhibiting the MDM2-p53 interaction (Novartis, 2022; Stein et al., 2022); and, (iii) alrizomadlin (APG-115, Ascentage), restores p53 expression by binding to MDM2 protein (Tolcher et al., 2019; Ascentage, 2022). Regarding the AKT1 finding, there are two small molecule drugs targeting the ATK1 p.E17K mutation being investigated: (i) capivasertib (AZD5363, AstraZeneca), inhibits all three isoforms of AKT by inhibiting downstream signaling of the AKT1 p.E17K mutation, (Chen et al., 2020; Kalinsky et al., 2021; AstraZeneca, 2022); and, (ii) BAY1125976 (Bayer), deactivates full-length AKT1 by binding into an allosteric binding pocket (Politz et al., 2017; Bayer, 2022) (see Supplementary Table 11).

To date, this study provides the most comprehensive analysis of a single human PSP neoplasm by utilizing radiomics, pathomics, and multiple genomic analyses. Using these studies insights are gleaned and discussed that span the patient’s initial presentation, tumor development with molecular determinants, and a precision medicine therapy plan is proposed in case of recurrence.

## Data Availability

The original contributions presented in the study are publicly available. The public study report page and summary-level phenotype data may be browsed at dbGaP: https://www.ncbi.nlm.nih.gov/projects/gap/cgi-bin/study.cgi?study_id=phs003154.v1.p1. The Individual-level data and sequence data are now available for download: https://www.ncbi.nlm.nih.gov/projects/gap/cgi-bin/study.cgi?study_id=phs003154.v1.p1. Data dictionaries and variable summaries are available on the dbGaP FTP site: https://ftp.ncbi.nlm.nih.gov/dbgap/studies/phs003154/phs003154.v1.p1/.
